# Endovascular Repair of Primary Thoracic Aortic Mural Thrombus Following Upper Limb Embolization

**DOI:** 10.21470/1678-9741-2020-0431

**Published:** 2021

**Authors:** Gianfranco Filippone, Gaetano La Barbera, Chiara Palermo, Fabrizio Valentino, Stefania Palimaru, Francesco Talarico

**Affiliations:** 1 Department of Vascular and Endovascular Surgery, ARNAS Civico Di Cristina Benfratelli, Palermo, Italy.

**Keywords:** Thrombosis, Aorta, Thoracic, Computed Tomography, Thromboembolism, Upper Extremity, Lower Extremity

## Abstract

We report the case of a 41-year-old female who presented with left upper limb embolization due to primary thoracic aortic mural thrombus; this latter represented an uncommon condition with difficult diagnosis and a high rate of life-threatening complications. Upper extremities embolization is extremely rare because it usually occurs in the lower limbs. Management strategy is still controversial, and no clear guidelines indicate superiority of either conservative or invasive treatment approach to date. Our report illustrates how endovascular exclusion of thoracic aortic mural thrombus has the advantage to be a low-risk procedure that represents a definitive therapy.

**Table:** 

Abbreviations, acronyms & symbols	
AULI	= Acute upper limb ischemia
CTA	= Computed tomography angiography
PTAMT	= Primary thoracic aortic mural thrombus
TEE	= Transesophageal echocardiography
TEVAR	= Thoracic aortic endovascular repair

## INTRODUCTION

Acute upper limb ischemia (AULI) is less common than acute lower limb ischemia. The annual incidence of AULI has been reported as 1.3 cases per 100,000 patients, accounting for 2% to 18% of surgical procedures for critical limb ischemia ^[1]^.

Over 80% of all peripheral and visceral emboli originate from disturbances of the cardiac function itself such as atrial fibrillation, myocardial infarction, endocarditis, and prosthetic heart valves. Noncardiac causes include aortic pathologies such as aneurysmal lesions, dissections, penetrating ulcers, or traumatic lesions. The amputation rate following acute limb ischemia is estimated at 13-14%, while mortality is at 9-12% ^[2]^.

Successful revascularization and limb salvage depend on timely diagnosis and localization of the arterial occlusion.

Since the initial description by Weismann and Tobin in 1958 ^[3]^, primary thoracic aortic mural thrombus (PTAMT) has been accepted as a definite clinical entity, developing in the absence of pre-existing aortic disease, and at the same time as an important source of noncardiogenic emboli ^[4]^. AULI as initial presentation is extremely rare, whereas the recurrence of thromboembolic events in patients which were managed by medical therapy alone was observed up to 34.6% of all cases ^[5]^.

Mortality and recurrent thrombus formation call into question the usefulness of surgical treatment over medical therapy and, to date, no clear guidelines indicate superiority of either conservative or invasive treatment approach ^[4]^. The patient provided written consent at the time of operation for her case and information to be used in research and publication.

## CASE REPORT

A 41-year-old female was referred because of acute ischemia of the left upper limb causing severe sensory loss and motor deficit. She was afebrile, in regular rhythm, smoker, and with family history of homocysteinemia. Considering clinical presentation, computed tomography angiography (CTA) was urgently scheduled. CTA images, excluding parietal infiltration, showed an intraluminal pedunculated filling defect arising from the isthmus of the aorta, the left subclavian artery occlusion, and a bovine aortic arch configuration (Figures 1A and 1B). Transthoracic echocardiography was negative for interatrial shunt or presence of thrombi in the left atrium and revealed a normal left ventricular function. Heparin drip was started with improvement of the symptomatology and recovery of neurologic disturbances, restored blood flow was checked by duplex ultrasound. Transesophageal echocardiography (TEE) showed the thrombus with a long distal free-floating pedicle segment (Figure 1C).

**Fig. 1A:**
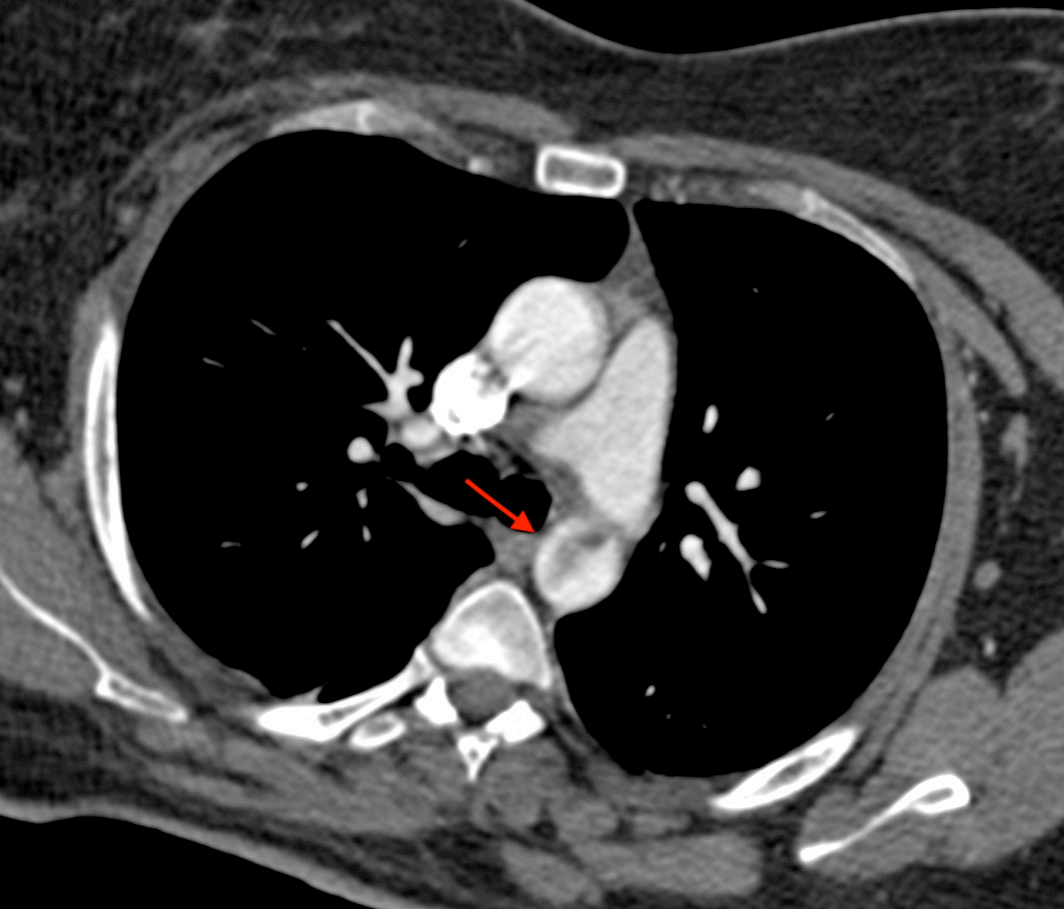
Computed tomography angiography axial scan showing the descending thoracic intraluminal aortic filling defect (red arrow).

**Fig. 1B:**
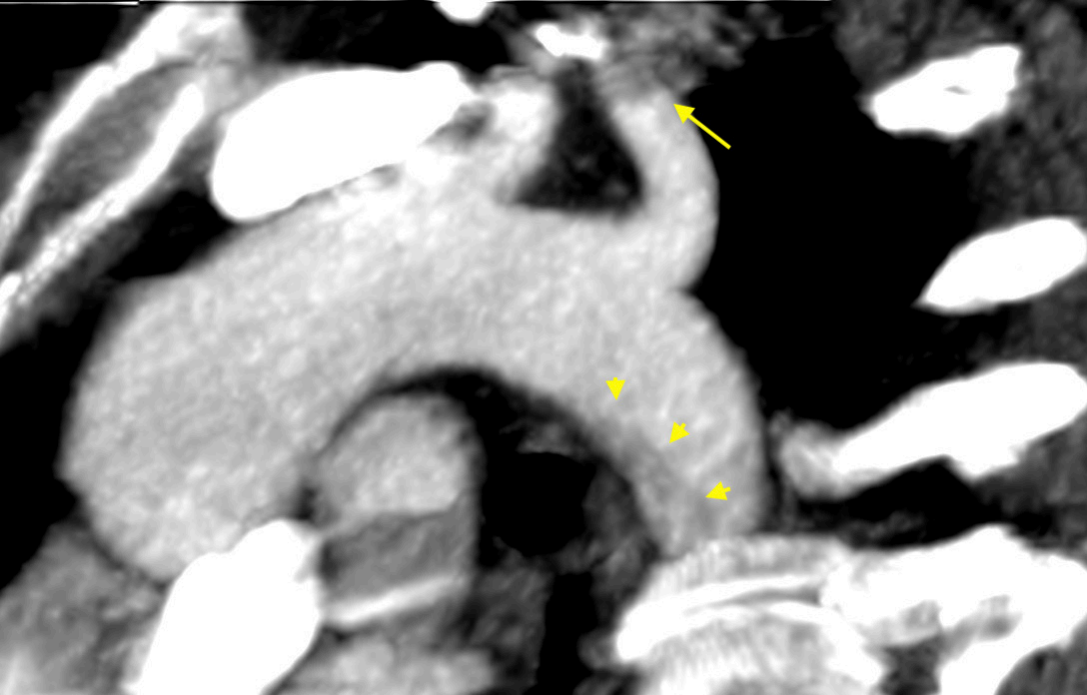
Volume-rendered two-dimensional imaging showing the left subclavian artery occlusion (yellow arrow) and the primary thoracic aortic mural thrombus (yellow arrowheads).

**Fig. 1C:**
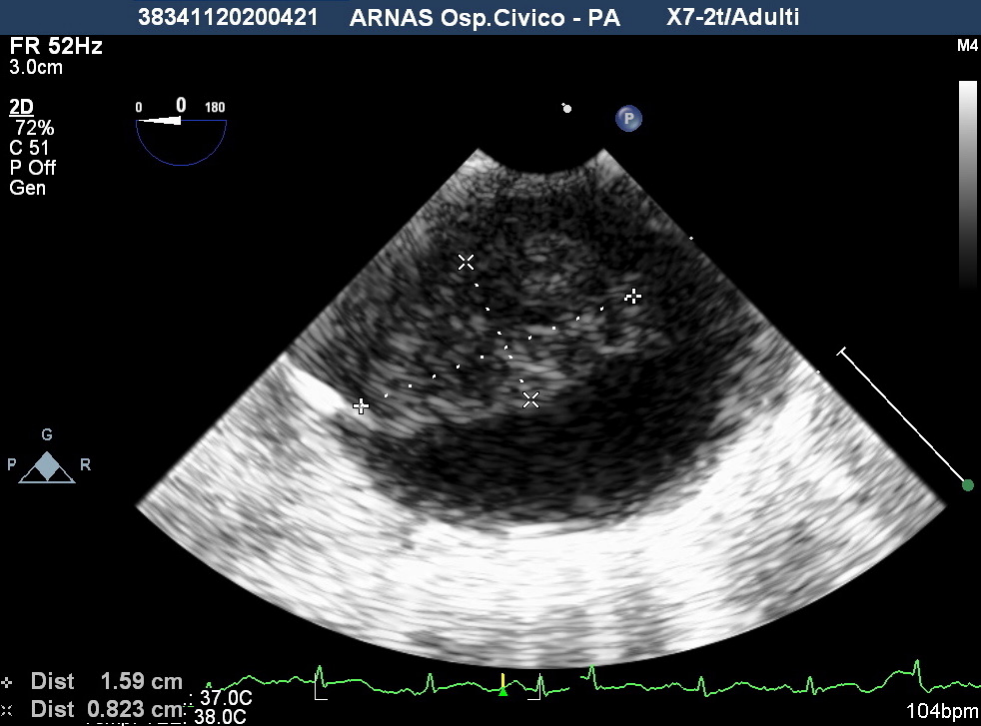
Transesophageal echocardiography showing aortic mural thrombus as pedunculated.

Ten days later, a follow-up TEE showed no modifications in thrombus dimension and the aortic team performed urgent thoracic aortic endovascular repair (TEVAR) as risk prevention of further embolic events (Figure 1D).

**Fig. 1D:**
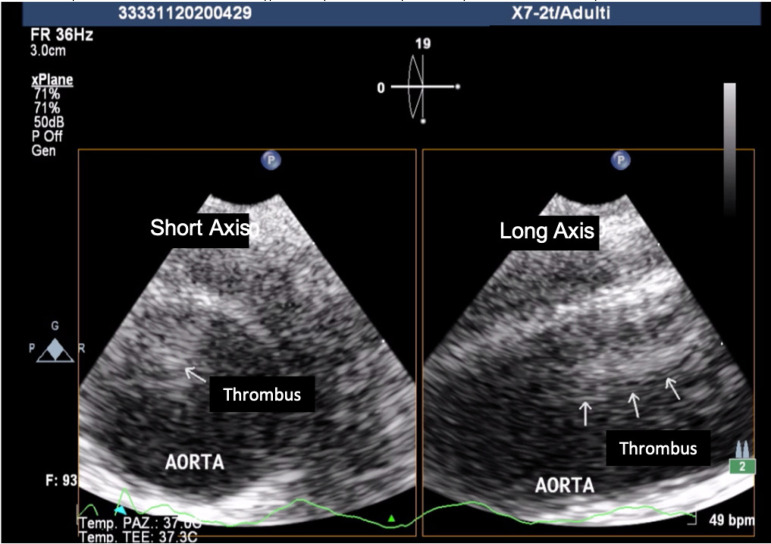
Follow-up transesophageal echocardiography showing no modifications of aortic mural thrombus in short axis (left) and long axis (right).

The procedure, planned with less than 10% in oversizing, was performed under general anesthesia in an operating room equipped with a portable C-arm (EuroColumbus, Milano, Lombardia, Italia). The right brachial artery was catheterized with a 6F 45-cm long introducer sheath for angiography (Figure 2A) and the right common femoral artery was surgically exposed for deployment of the device.

**Fig. 2A:**
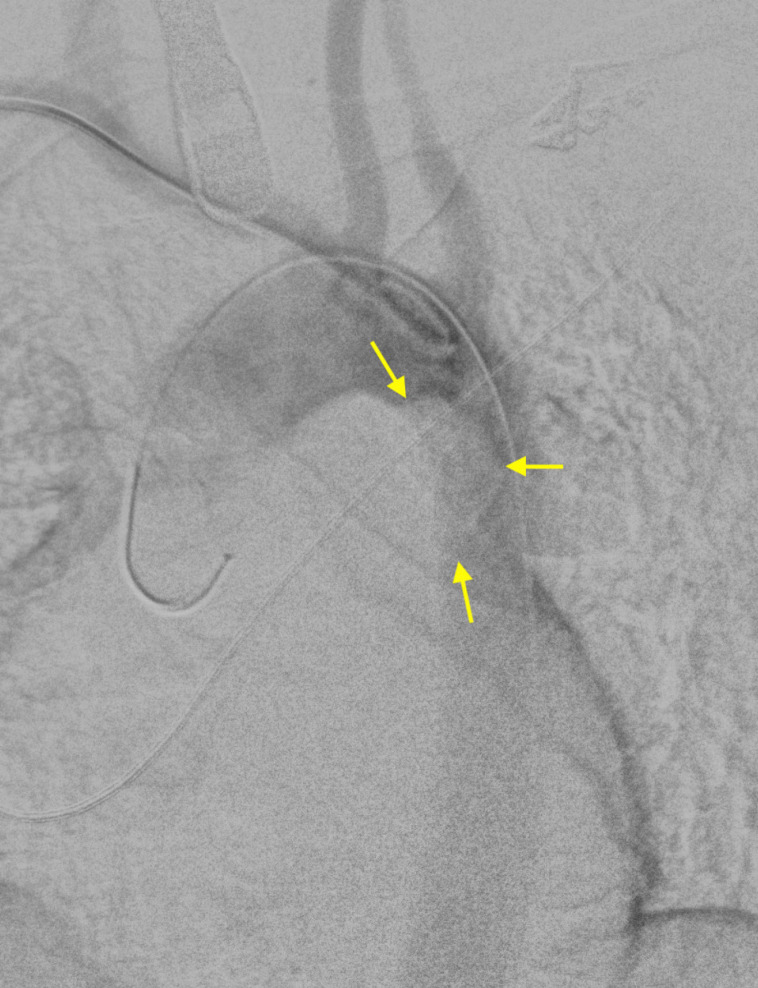
Angiogram showing the intra-aortic filling defect (yellow arrow).

TEE was utilized to monitor the cardiac function as well as the positions of the wires at the thoracic level, the deployment of the device, and the final outcome. The aorta was catheterized with a 5F pigtail catheter that was carefully advanced. Once the tip of the pigtail catheter reached the ascending aorta, a Lunderquist extra-stiff guidewire (Cook Medical, Bloomington, Indiana, United States of America) was inserted and parked adjacent to the aortic valve plane. A 24 mm × 105 mm Zenith Alpha^TM^ Graft (Cook Medical, Bloomington, Indiana, United States of America) was introduced and deployed in zone 2 (Figure 2B). Final outcome was checked by angiogram and TEE (Figure 2C).

**Fig. 2B:**
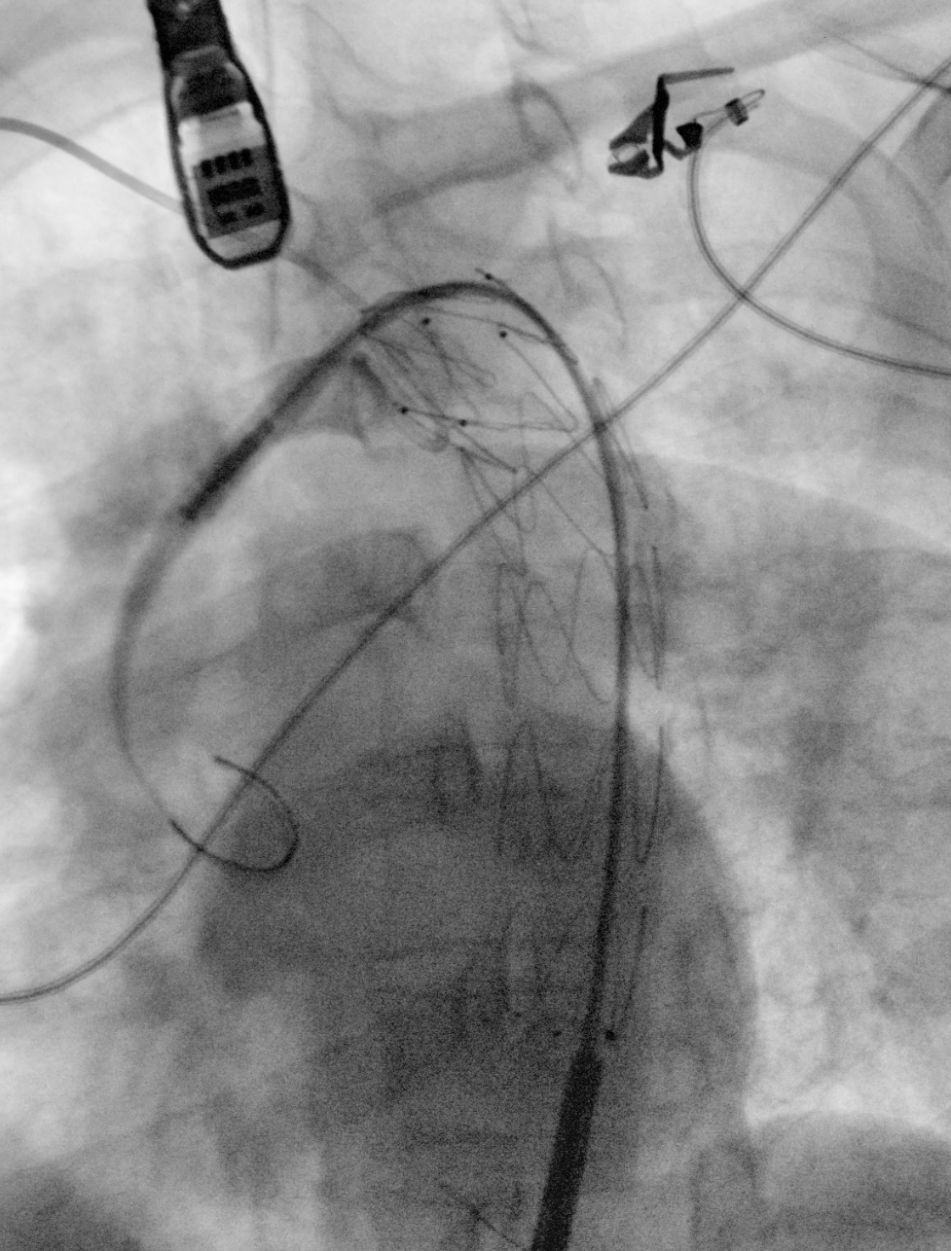
Angiogram showing graft deployment with descending thoracic aortic thrombus exclusion.

**Fig. 2C:**
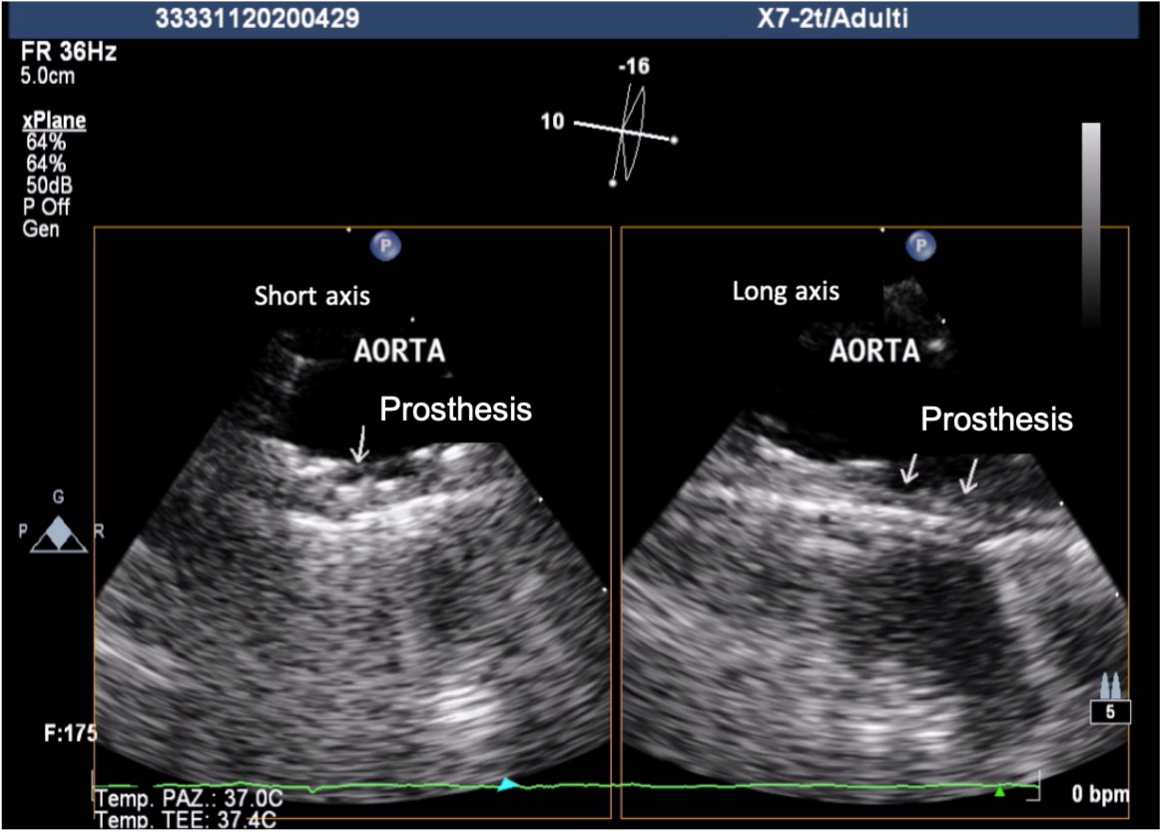
Intraoperative transesophageal echocardiography showing complete exclusion of aortic mural thrombus after endograft deployment. Short axis (left) and long axis (right).

Postoperative course was uneventful and on the 5^th^ postoperative day the patient was discharged with prescription of lifelong anticoagulation. Six months later, follow-up CTA confirmed the complete exclusion of PTAMT (Figure 2D).

**Fig. 2D:**
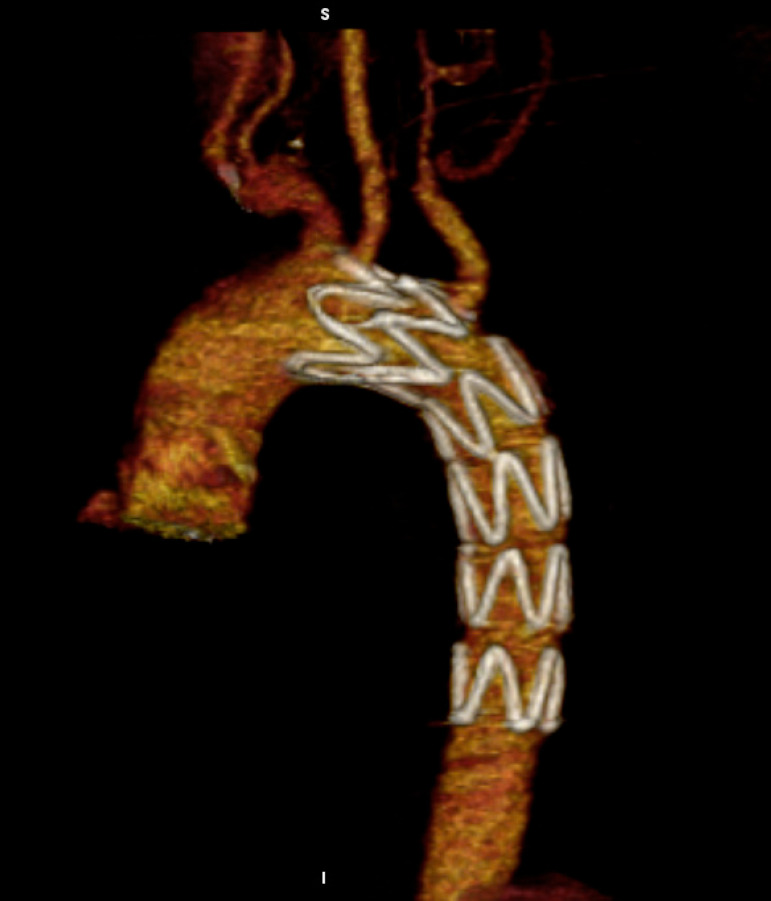
Volume-rendered three-dimensional follow-up computed tomography angiography imaging showing the endograft deployment in zone 2 and the exclusion of primary thoracic aortic mural thrombus.

## COMMENTS

PTAMT is an uncommon condition with a difficult diagnosis and a high rate of complications, including high mortality, developing in the absence of pre-existing aortic disease ^[6]^. The pathophysiology of thrombus formation in a macroscopically normal aorta is not well understood to date; a correlation with underlying disease as hypercoagulable disorders (*i.e*., homocysteinemia) has been suggested ^[2]^. Our patient had only history of hypercoagulopathy state, but the presence of a bovine aortic arch configuration and the location at isthmus suggested both issues as “*locus minoris resistentiae*”, and therefore as injury-prone areas.

The patient’s tobacco addiction contributed to lower the positive effect of estrogen on lipid profiles, this side effect is particularly effective in women because of endothelial damage and impairment of the release of nitric oxide, a substance important for inhibiting platelet aggregation, smooth-muscle-cell proliferation, and monocyte adhesion. In addition, smoking increases blood viscosity via red blood cell aggregation and elevations in fibrinogen levels ^[7]^.

PTAMT incidence, clinical course, prognostic implications, and optimal therapy have not been well established and pose a clinical challenge ^[8]^.

Autopsy reports quote a 0.45% incidence in the general population, with 17% showing evidence of distal embolization: 56.8% to lower extremities, 32.4% to visceral organs, 4.05% were cerebral emboli, and only 1.35% of the patients presented with upper extremity emboli ^[2]^.

Furthermore, upper extremity embolization as initial presentation is unusual - only three cases to our knowledge are reported and all of them have been treated medically ^[5,6]^.

Treatment strategies have largely been dependent on anatomic location as well as on morphologic features of the thrombus and, to date, no clear guidelines indicate superiority of either conservative or invasive treatment approach ^[4]^.

However, the recurrence of aortic thrombi in patients receiving medical therapy alone represent a major concern. This subset of patients showed a 34,6% of persistence rate and mostly had recurrence within two to four weeks; furthermore, the presence of mobile lesions showed a 30-fold greater risk of distal embolization causing end-organ damage and/or limb ischemia ^[9]^.

Moreover, a 73% incidence of embolic events is reported among patients with pedunculated and highly mobile aortic thrombi, as opposed to only 12% when thrombus was layered and immobile ^[6]^. A TEE-based study of protruding aortic plaques showed that the presence of mobile lesions was linked to 30-fold increased risk of distal embolization and recurrent embolism significantly increases the risk of major amputation (9% for anticoagulation alone vs. 2.3% for surgical group) and life-threatening visceral ischemia ^[9,10]^. In our patient, mural thrombus was pedunculated with a 20-mm long free-floating segment cause of increasing risk of embolization. The systematic evaluation of thrombus mobility provided by a real-time dynamic imaging, notably TEE, is necessary to identify patients with high risk of embolism. Such patients can be considered as good candidates for early endovascular treatment given the potential failure risk of a purely conservative approach ^[11]^.

TEVAR for mural thrombi of the descending aorta was first described in 2004 ^[12]^, since then endovascular repair has been increasingly described to treat PTAMT ^[2]^. Endovascular repair has the advantage of being minimally invasive, presenting low risk and representing a successful definitive therapy for PTAMT. In a small series, there has been no difference in the outcomes between stent graft and bare-metal stent implantation. The risk of distal embolism during wire manipulation and stent-graft deployment could represent a limitation of endovascular treatment but no such complications were described in the reported cases ^[2,5]^.

Several measures were suggested to prevent the risk of distal embolization. Firstly, the procedure must be performed without interrupting systemic anticoagulation. Secondly, manipulation of wires and catheters in the aorta must be minimized. Thirdly, an appropriate stent graft has to be chosen in terms of diameter (oversizing < 10%) and length making it possible to cover the aorta at least 2 cm above and below the implantation site of the thrombus ^[11]^..

A major issue, in our opinion, may be the risk of endograft infolding; it has been speculated a correlation with stent graft oversizing and in this regards, we have adopted only 10% in stent graft oversizing ^[13]^.

## CONCLUSION

PTAMT, despite uncommon or probably underestimated, must be considered in the differential diagnosis of embolic events, otherwise only the epiphenomenon of an undetected disease would be treated. Mortality and recurrent thrombus formation call into question the usefulness of surgical treatment over medical therapy. We consider, according with several authors ^[2,10,11]^, the presence of a mobile thrombus as a relative indication for primary intervention. Moreover, the risk/benefit ratio favors, in our opinion, the endovascular repair in order to avoid the potential catastrophic consequences of peripheral and/or visceral embolization.

There is a need for more detailed studies and better follow-up regarding the use of TEVAR for PTAMT to report on the durability of results from such treatment modality.

**Table:** 

Authors' roles & responsibilities	
GF	Substantial contributions to the conception or design of the work; or the acquisition, analysis, or interpretation of data for the work; drafting the work or revising it critically for important intellectual content; final approval of the version to be published
GLB	Substantial contributions to the conception or design of the work; or the acquisition, analysis, or interpretation of data for the work; drafting the work or revising it critically for important intellectual content; final approval of the version to be published
CP	Substantial contributions to the conception or design of the work; or the acquisition, analysis, or interpretation of data for the work; drafting the work or revising it critically for important intellectual content; final approval of the version to be published
FV	Substantial contributions to the conception or design of the work; or the acquisition, analysis, or interpretation of data for the work; drafting the work or revising it critically for important intellectual content; final approval of the version to be published
SP	Substantial contributions to the conception or design of the work; or the acquisition, analysis, or interpretation of data for the work; drafting the work or revising it critically for important intellectual content; final approval of the version to be published
FT	Substantial contributions to the conception or design of the work; or the acquisition, analysis, or interpretation of data for the work; drafting the work or revising it critically for important intellectual content; final approval of the version to be published
